# Portal vein thrombosis in a 10-month-old infant as a complication of neonatal umbilical catheterization: a case report

**DOI:** 10.1097/MS9.0000000000001173

**Published:** 2023-08-09

**Authors:** Sultaneh Haddad, Salim Haddad, Komait Swaid, Nafiza Martini, Marah Mansour, Lina Alkouri

**Affiliations:** a Faculty of Medicine, Aleppo University, Aleppo; b Faculty of Medicine, Damascus University; c Stemosis for Scientific Research; d Damascus University, Pediatric University Hospital, Damascus; e Faculty of Medicine, Tartous University, Tartous, Syrian Arab Republic.; f Department of Surgery, Division of Colon and Rectal Surgery, Mayo Clinic, Rochester, Minnesota, USA

**Keywords:** case report, esophageal varices, octreotide, portal vein thrombosis, sclerotherapy, umbilical catheterization

## Abstract

**Introduction:**

Esophageal varices bleeding after portal hypertension is a rare condition in children but is associated with significant morbidity and mortality. Neonatal umbilical catheterization is one of the risk factors for the development of portal vein thrombosis (PVT) and portal hypertension.

**Case presentation:**

Neonatal umbilical catheterization was used here to provide appropriate treatment for postpartum sepsis. Color Doppler revealed an approximate total obstruction of the portal vein, and endoscopy showed esophageal varices. The patient was sequentially managed with endoscopic sclerotherapy.

**Discussion:**

The manifestations associated with PVT (like splenomegaly and bleeding esophageal varices) have been detected in a 10-month-old, which is considered a relatively young age according to the medical literature review.

**Conclusion:**

Using umbilical vein catheterization in neonates may be associated with several complications, including PVT. This case report describes a rare instance of portal hypertension complicated by bleeding esophageal varices in a 10-month-old infant who had undergone newborn umbilical catheterization.

## Introduction

Highlights

- Esophageal varices bleeding after portal hypertension is a rare condition in children but is associated with significant morbidity and mortality.
- Neonatal umbilical catheterization is one of the risk factors for the development of portal vein thrombosis and portal hypertension.
- Doctors should think of portal vein hypertension as a differential diagnosis for children who come with upper gastrointestinal bleeding.
- This case aims to increase awareness of the possible complications of umbilical vein catheterization through follow-up imaging.

Portal vein thrombosis (PVT) is the most important cause of extrahepatic portal hypertension and upper gastrointestinal bleeding in children. Other familiar causes of upper gastrointestinal bleeding in children are erosive esophagitis, peptic ulcers, Mallory–Weiss syndrome, and Henoch–Schonlein purpura^1,2^. One of the most common causes of portal hypertension in children is extrahepatic portal vein obstruction. Extrahepatic PVT could be sudden or gradual. The vast majority of patients will have splenomegaly upon physical examination. Bleeding from gastroesophageal varices is the most important complication and the main cause of death among children^3^. Specific neonatal events are identified in patients with PVT, such as sepsis, abdominal surgery, or umbilical vein catheterization (UVC). The incidence of thrombosis as a complication of UVC in the medical literature is 44%. Dehydration has also been described as a part of PVT development^4^. Documenting the echogenic intraluminal thrombus on grayscale ultrasound and the lack of the flow on color Doppler ultrasound pictures allow for the diagnosis of a portal venous thrombus to be made^5^. This case reports bleeding emesis from esophageal varices, managed with endoscopic sclerotherapy as well as the appropriate supportive therapy, and the bleeding was controlled successfully. The work has been reported in line with the SCARE (Surgical CAse REport) 2020 criteria^6^.

## Case presentation

**Figure 1:**
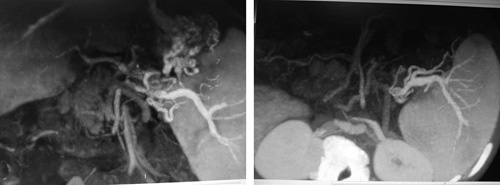
Esophagogastroduodenoscopy shows esophageal constriction stenosis caused by multiple esophageal ulcers, and esophageal varices (IV degree).

**Figure 2:**
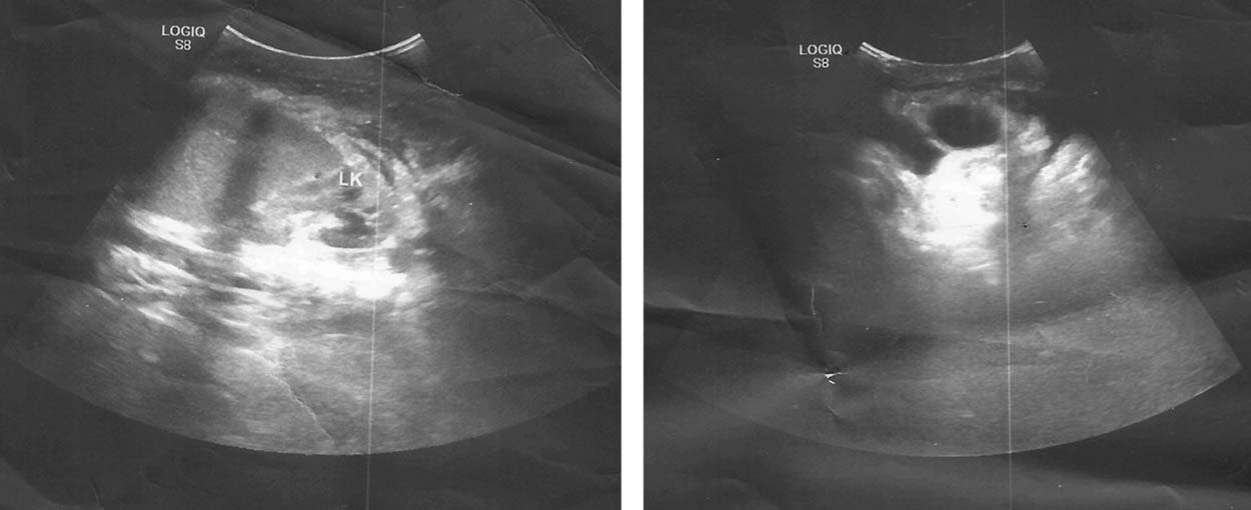
Ultrasound shows ascites and homogeneous splenomegaly.

**Figure 3:**
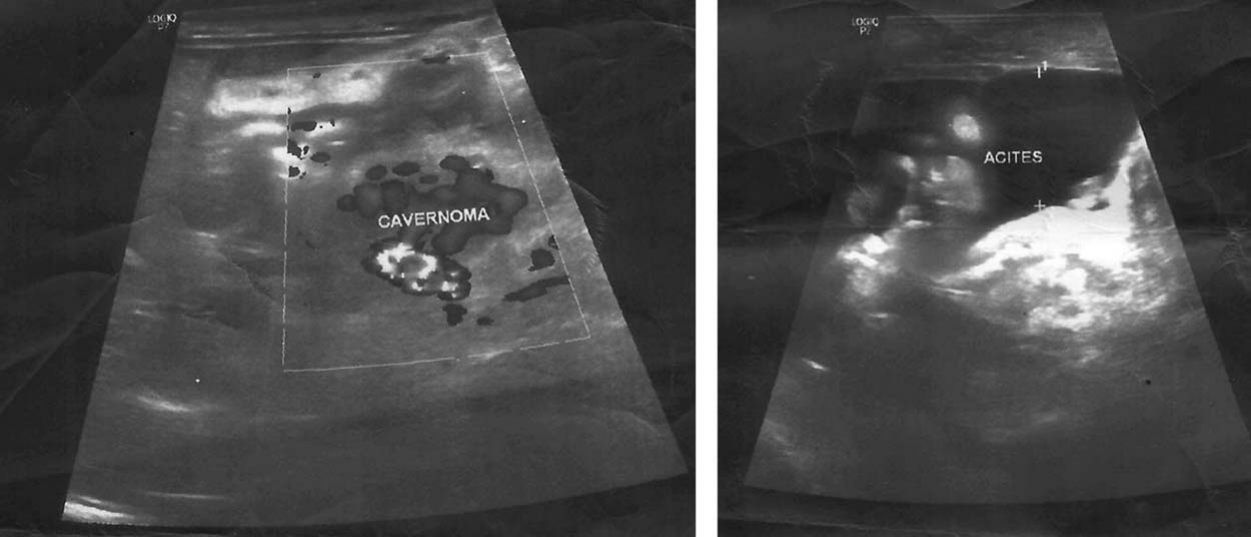
Doppler ultrasound shows impaired blood flow in the portal vein and varices in both the gastric antrum and splenic hilum.

**Figure 4:**
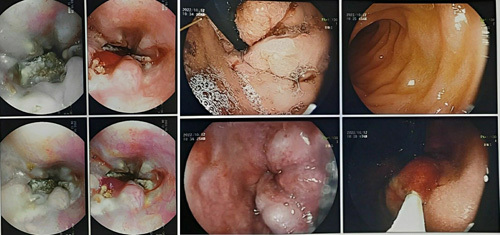
Multi-slice computer tomography scan shows total obstruction in the portal vein after the joining of the splenic and superior mesenteric veins.

A 10-month-old female patient presented to the Pediatrics Department with melena, and bloody vomitus, accompanied by fever and dehydration. Four episodes of bloody vomiting were reported prior to the presentation. Medical history includes sepsis 3 days after birth, treated with exchanged blood transfusions via an umbilical catheter, meningitis, which had also been treated, and abdominal distention following each breastfeeding, for which it was received in the incubator department in the hospital, and then it was transferred to bottle feeding. The parents also reported frequent episodes of pyrexia, which respond to antipyretics, in addition to frequent respiratory infections. The patient’s parents were consanguineous, but no family history or medication history was recorded. The physical examination revealed pyrexia (temperature: 39°C), sunken eyes due to dehydration, generalized mild jaundice color of the skin, enlarged abdomen, navel bulge, bilateral inguinal hernia, and an enlarged spleen that could be palpated 4–5 cm below the left costal margin, but no liver enlargement. Laboratory findings demonstrated the following, as shown in Table 1. Esophagogastroduodenoscopy showed esophageal constriction stenosis caused by multiple esophageal ulcers, esophageal varices (IV degree), and congestive gastritis (Fig. 1). Abdomen and groin ultrasound showed ascites measuring about 3 l, homogeneous splenomegaly, and an invisible portal vein in the hepatic hilum (Fig. 2). Doppler ultrasonography further revealed previous thrombosis in the splenic vein, splenomegaly, varices in both the gastric antrum and splenic hilum, fibrosis, and impaired blood flow in the portal vein furthermore (Fig. 3). Multi-slice computed tomography (MSCT) showed total obstruction in the portal vein after the joining of the splenic and superior mesenteric veins, ascites that fill both the abdomen and the pelvic areas, splenomegaly (9×7×5 cm), and retroperitoneal swollen lymph nodes (Fig. 4). Blood levels of antiphospholipid antibody, AT-III, protein S, protein C, and homocysteine were normal. The patient underwent sclerotherapy and paracentesis for ascites and was provided with a blood transfusion and supportive intravenous fluids. Octreotide (1 μg/kg/h), proton pump inhibitors (omeprazole), and antibiotics (vancomycin) were administered. Considering the follow-up, the patient was left on (linezolid), low doses of low-molecular-weight heparin (LMWH), propranolol, and regular ultrasonic and endoscopic follow-up were recommended. The patient was discharged one-week post-operation in good general condition.

**Table 1:** Laboratory results are as follows.

Test	Value
WBC	41 300/mm^3^
CRP	16.5 mg/l
Platelets	37×10^3^/μl
Albumin	2.7 g/dl
BUN	74 mg/dl
Hemoglobin	6.8 g/dl
Potassium (K)	3.1 mEq/l

BUN, blood urea nitrogen; CRP, C-reactive protein; WBC, white blood cells.

## Discussion

A pressure gradient of more than 5 mmHg between the portal vein and inferior vena cava characterizes portal hypertension. One of the causes of portal hypertension in children is PVT which is a rare disorder with an incidence of 1 in 100 000 lives^7^.

The term ‘PVT’ describes a complete or partial blockage of the blood flow in this area as a result of thrombus formation. Balfour and Stewart initially detailed the PVT case in 1868. Although the exact causes of PVT in children are unknown, there are a number of risk factors that are listed. These are divided into two main groups: general causes (procoagulant status) and local factors (abdominal infections, abdominal surgery, and umbilical catheter)^7^.

These catheters are used for the administration of parenteral medication and nutrition. However, they have complications like infection, PVT, hepatic or vascular injury, arrhythmia, and sepsis^8^. In our case, the umbilical catheter is used for the administration of parenteral medication for sepsis treatment, and we suspect that it caused the PVT after denying any thrombotic disorder based on laboratory values of blood analyses.

In the study of Maamouri *et al*.^9^, the mean age of the children with PVT due to UVC was 33.1±3.55 months. Most PVT instances are detected in childhood because they rarely result in clinical manifestations during the neonatal period. Our case with blatant clinical manifestations was diagnosed during the neonatal period (10 months).

The most common manifestations of PVT were upper gastrointestinal bleeding and splenomegaly. Hepatomegaly and ascites were also detected. In general, laboratory testing for aminotransferases, albumin, and coagulation factors shows normal results. Following episodes of upper bleeding, albumin may decrease and be accompanied by ascites^10^. All these clinical manifestations associated with PVT were detected in our case, in addition to bilateral inguinal hernia. Hypoalbuminemia, thrombocytopenia, and low hemoglobin count were also detected.

Noninvasive methods used for diagnosing PVT include real-time or duplex Doppler sonography, computed tomography, or MRI^11^. In this study, Doppler sonography and MSCT were used for diagnosis. For the diagnosis of esophageal and gastric varices, upper gastrointestinal endoscopy is the gold standard^1^.

For the treatment of PVT, LMWH is advised by current guidelines followed by vitamin K antagonists^12^. In our patient, the treatment with heparin or other anticoagulant was contraindicated due to the bleeding esophageal varices which she came with.

Transjugular intrahepatic portosystemic shunt (TIPS) may be considered in cases of occlusive PVT with worsening portal hypertension and esophageal varices. A decision to pursue TIPS should be carefully considered in a multidisciplinary manner in light of the high prevalence of hepatic encephalopathy and low patency rates^13^. The decision to operate carries a high risk of mortality for the child. This type of operation has not been performed before in Syria. Therefore, it was excluded at a time symptoms had regressed on conservative treatment.

Endoscopic sclerotherapy is used, where a flexible catheter with a needle tip is used to inject a sclerosing substance into the variceal lumen, causing thrombosis of the vessel and inflammation of the tissues around it. However, several local (ulcers, strictures) and systemic (fever, bacteremia) complications may arise after sclerotherapy^14^.

Somatostatin analogs are administered intravenously to both adult and pediatric patients with portal hypertension as a conventional treatment for acute gastrointestinal bleeding episodes and acute bleeding that is not controlled by sclerotherapy and banding^15,16^. Massive splenomegaly, recurrent bleeding despite proper endoscopic treatment call for surgical intervention^10^. In our case, treatment of the varices depended on using octreotide and sclerotherapy without complications. Surgery was not required because the symptoms had regressed, and the enlargement of the spleen had decreased based on only conservative treatment.

## Conclusion

This case aims to increase awareness of the possible complications of neonatal UVC, including PVT, which results in portal hypertension and esophageal varices. We suggest doing follow-up imaging of the catheter position immediately after placement and during hospitalization to prevent future PVT in neonates who underwent UVC.

## Ethics approval

Ethical approval for this study was provided by the Ethical Committee of Damascus University, Damascus, Syria, on 17July 2022, with number 4019.

## Consent

Written informed consent was obtained from the patient’s parent for the publication of this case report and any accompanying images. A copy of the written consent is available for review by the Editor-in-Chief of this journal.

## Sources of funding

Not applicable.

## Author contribution

S.H. is the first author: contributed to drafting, reviewing and editing, bibliography, and approved the final manuscript; S.H. is a co-first author: contributed to drafting, reviewing and editing, bibliography, and approved the final manuscript; K.S.: contributed to drafting, reviewing and editing, bibliography, and approved the final manuscript; N.M.: contributed to corresponding, reviewing, editing, and approved the final manuscript; M.M.: contributed to reviewing, editing, and approved the final manuscript; L.A.: contributed to reviewing, editing, supervising, and approved the final manuscript

## Conflicts of interest disclosure

The authors declare that they have no conflicts of interest.

## Research registration unique identifying number (UIN)

Not applicable.

## Guarantor

Dr Lina Khouri.

## Provenance and peer review

Not commissioned, externally peer-reviewed.

## Data availability statement

Not applicable.
